# The effects of resveratrol on endothelial progenitor cells and apoptosis biomarkers in postmenopausal women with chronic coronary heart disease: a randomized controlled trial

**DOI:** 10.3389/fnut.2026.1814668

**Published:** 2026-05-14

**Authors:** Gustavo Henrique Ferreira Gonçalinho, Verônica Coelho, Sandra Maria Monteiro, Célia Maria Cassaro Strunz, Jorge Kalil, Antonio de Padua Mansur

**Affiliations:** 1 Disciplina de Cardiologia, Departamento de Cardiopneumologia, Faculdade de Medicina, Universidade de São Paulo, São Paulo, Brazil; 2 Serviço de Prevenção e Reabilitação Cardiovascular, Instituto do Coração (InCor), Hospital das Clínicas HCFMUSP, Faculdade de Medicina, Universidade de São Paulo, São Paulo, Brazil; 3 Laboratório de Imunologia, Instituto do Coração (InCor), Hospital das Clínicas HCFMUSP, Faculdade de Medicina, Universidade de São Paulo, São Paulo, Brazil; 4 Instituto de Investigação em Imunologia do Instituto Nacional de Ciências e Tecnologia (III-INCT), São Paulo, Brazil; 5 Laboratório de Análises Clínicas, Instituto do Coração (InCor), Hospital das Clínicas HCFMUSP, Faculdade de Medicina, Universidade de São Paulo, São Paulo, Brazil; 6 Disciplina de Imunologia Clínica e Alergologia, Departamento de Clínica Médica, Faculdade de Medicina, Universidade de São Paulo, São Paulo, Brazil

**Keywords:** apoptosis, coronary artery disease, endothelial function, endothelial progenitor cells, menopause, resveratrol

## Abstract

**Background and aims:**

Menopause is an important cardiovascular risk factor in women likely due to marked estrogen decline. Estrogens affect key processes in atherosclerosis, maintaining endothelial integrity by inhibiting endothelial apoptosis and promoting regeneration through endothelial progenitor cells (EPCs). Phytoestrogens, such as resveratrol, have therapeutic potential for women’s cardiovascular health. The present study aimed to determine whether resveratrol modulated circulating apoptosis biomarkers and EPCs in postmenopausal women with chronic coronary heart disease (CHD).

**Methods:**

Twenty postmenopausal women with chronic CHD were allocated to an intervention group receiving 1,000 mg of resveratrol or placebo for 90 days. Circulating markers of apoptosis (caspases 3 and 9, survivin, cIAP2, XIAP, Bcl-2, cytochrome c, sTNFR2, and S100A12), SIRT1 and 3, cytokines and EPCs were assessed.

**Results:**

At the end of the study, the resveratrol group showed an increase in serum cIAP2 (*p* = 0.018) and Bcl-2 (*p* = 0.043), and a decrease in caspase-9 (*B* = −7.152, 95%CI = −13.554 to −0.751) and in circulating CD34+/KDR+ (*B* = −0.200, 95%CI = −0.304 to −0.096). In addition, XIAP levels were lower than placebo group (*p* = 0.047). No significant differences were observed for sirtuins, cytokines, lipid profile, glucose, and blood pressure.

**Conclusion:**

Resveratrol increased serum Bcl-2 and cIAP2, and also reduced caspase 9 and circulating EPCs in postmenopausal women with chronic CHD, likely unrelated to circulating SIRT1 and SIRT3. The effects of resveratrol treatment in this population group should be assessed with caution, as a reduction in EPCs is associated with impaired endothelial function and an increased risk of cardiovascular disease.

## Introduction

Cardiovascular diseases (CVD) are the leading cause of death in women worldwide, especially after menopause, a period characterized by a decline in estrogen levels and subsequent amenorrhea (1). The onset of CVD occurs approximately 7–10 years later in women than in men, and this delay is mainly attributed to the vascular protection provided by estradiol during the fertile period (2–5). After the decline in estrogen levels in menopause, the higher CVD incidence and risk found in this population were associated with structural and functional alterations in cardiovascular system, suggesting a key role of estrogens (6). The main CVD associated with deaths and loss of quality of life in this group is coronary heart disease (CHD), which has atherosclerosis as its pathophysiological basis and a multifactorial etiology (7).

**Figure 1:**
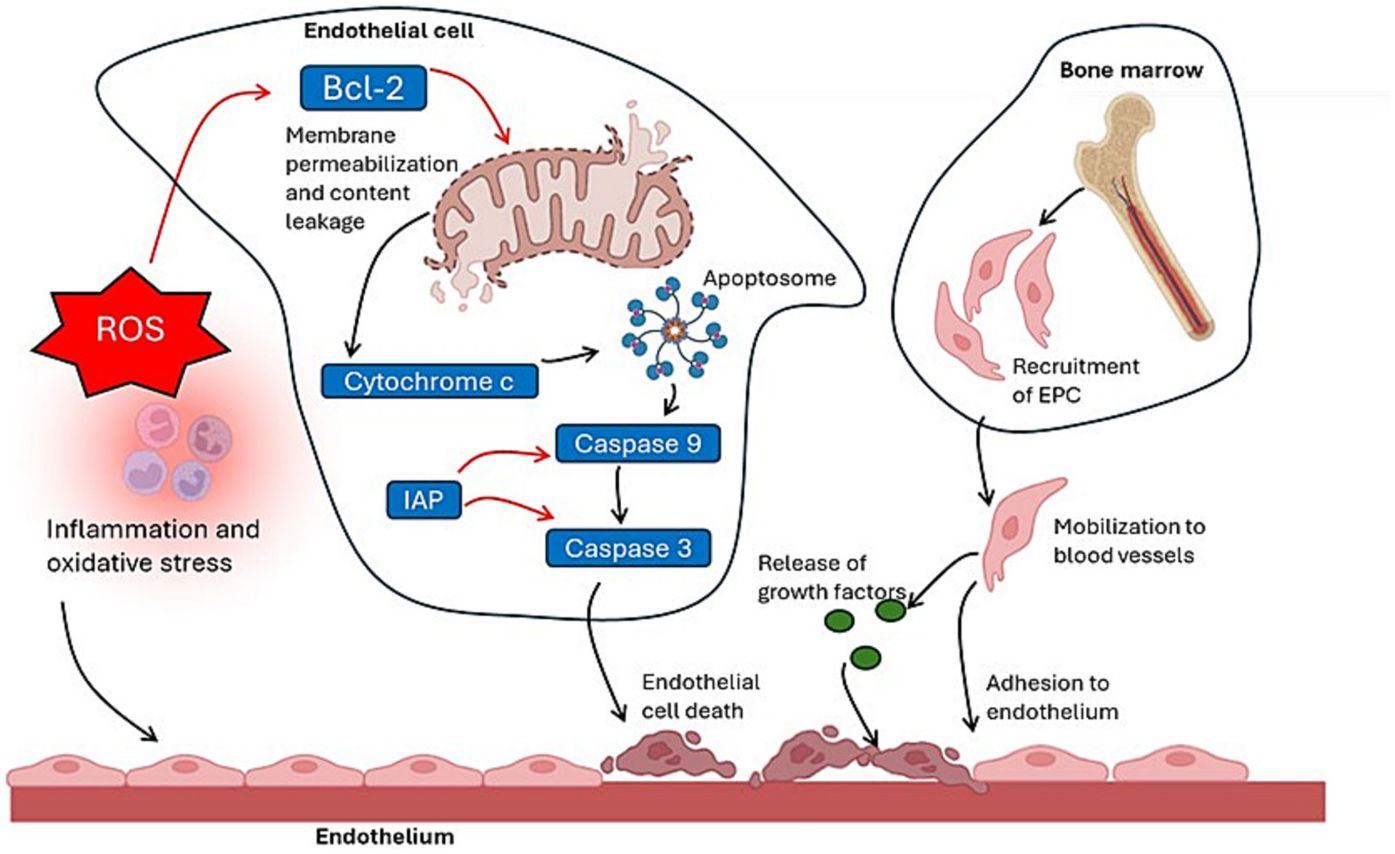
Effects of inflammation and oxidative stress on intrinsic apoptosis pathway of endothelial cells and recruitment of endothelial progenitor cells for vascular repair (red arrows = inhibition; black arrows = induction). Bcl-2, B-cell lymphoma 2; EPC, endothelial progenitor cells; IAP, inhibitor of apoptosis proteins; ROS, reactive oxygen species.

It has been recognized that apoptosis is a key process in the pathogenesis of atherosclerosis and CVD, however, the extent of this role remains unclear. Studies showed that significant apoptosis of cardiomyocytes is present in lesions of heart tissue in patients with various CVD (8, 9). Apoptosis is a regulated program of cell death and is activated in cardiomyocytes by factors present in CVD, such as oxidative stress, DNA damage, and inflammation (10). There are three signaling pathways to induce apoptosis: the extrinsic pathway, which is mediated by the death receptors and ligands such as Fas/FasL and TNF-α/TNFR, and activation of caspase 8; the intrinsic pathway, which involves mitochondrial dysfunction, cytochrome c release, formation of the apoptosome complex and activation of caspase 9; and the pathway activated by endoplasmic reticulum (ER) stress and activation of caspase 12 (11). The activation of caspases can be blocked by the anti-apoptotic proteins inhibition of apoptosis proteins (IAPs) and Bcl-2, which are mainly involved in the intrinsic apoptotic pathway (11) (Figure 1).

The presentation of apoptosis differs among CVD diseases. In acute myocardial infarction (AMI), cell death caused by ischemia/reperfusion injury resulting from coronary occlusion is the central event in pathogenesis, and apoptosis is the main type of cell death in the disease (12, 13). In heart failure, apoptosis is also found in cardiomyocytes, but to a lesser extent (13). In atherosclerosis, apoptosis of endothelial cells may be an early event which promotes the formation and development of lesions, characterized by the accumulation of cellular debris from the apoptotic process, cholesterol, and a calcified matrix which trigger inflammatory stimuli and that narrows the arterial lumen (14). Apoptosis of vascular smooth muscle cells (VSMCs) is also found in advanced atherosclerotic plaques and leads to fibrous cap thinning and promotes necrotic core formation and calcification, causing plaque instability and increased risk (15). Efferocytosis of apoptotic cells prevents the release of pro-inflammatory cell lysate and resolves inflammation, however, it has been found that in advanced lesions, efferocytosis signaling is found reduced, as apoptosis signaling is increased, causing the inflammatory stimulus to persist in the lesions (14). Increased circulating levels of pro-apoptotic markers are found in individuals at high cardiovascular risk and with metabolic syndrome (16, 17), acute myocardial infarction (AMI) and unstable angina pectoris (18), worsened left ventricular function (19), and heart failure (20), suggesting ongoing elevated apoptosis signaling. Studies *in vitro* and in animals showed that estrogen prevents endothelial cell apoptosis (21–23), and hormone therapy with 17β-estradiol (E2) and norethisterone decreased the apoptotic protein levels of FasL in postmenopausal women, suggesting a link between estrogens, apoptosis, and increased cardiovascular risk in this group (24). Therefore, inhibition of apoptosis may be useful for the treatment of CVD in postmenopausal women (10, 25, 26).

As apoptosis increases in CVD and deteriorates the endothelial and vascular functions, the stimulation of endothelial regeneration through endothelial progenitor cells (EPCs) as a strategy has gained prominence (27) (Figure 1). EPCs are a heterogeneous group of cells derived from bone marrow and are present in peripheral blood mononuclear cells (PBMC) at different stages of endothelial differentiation, having vascular tropism effect that is involved in repair of endothelial damage (27). EPCs levels have been associated with endothelial function measured by flow-mediated vasodilation (FMD) (28), inversely associated with risk factors for CHD (29), and has also been shown to predict CVD outcomes, which suggests a cardioprotective effect of EPCs (30, 31). Furthermore, studies showed that estrogens induce angiogenesis through EPCs (32, 33), and EPCs levels are found to be reduced in postmenopausal women (34).

Several strategies to apoptosis inhibition and increase circulating EPCs have been studied for potential CVD treatment. A compound with high therapeutic potential for CVD is resveratrol, a polyphenol naturally found in grapes, cocoa, strawberries, tomatoes, peanuts, soybeans, hops, blackberries, raspberries, and blueberries (35). Among the effects of resveratrol on cardiovascular health, the most notable are the anti-inflammatory, antioxidant, and angio-regulatory effects against atherosclerosis, ischemia, and cardiomyopathy (36). Many of these effects are linked to activation of sirtuins (SIRT), especially SIRT1 (37). SIRT are NAD^+^-dependent histone deacetylase (HDAC) that modulate several metabolic and physiologic processes, but importantly, they inhibit apoptosis and modulate endothelial function, especially SIRT1 and SIRT3 (38–40). However, there is a lack of randomized clinical trials assessing the effects of resveratrol on sirtuins and their potential relation with apoptosis inhibition and endothelial function – particularly circulating EPCs. Therefore, this study aimed to explore the effects of resveratrol supplementation treatment on circulating EPCs and apoptosis biomarkers of postmenopausal women with CHD.

## Methods

### Study design and participants

In this randomized, double-blind, placebo-controlled trial, 20 post-menopausal women aged ≥ 55 years old with stable chronic CAD were recruited from the Heart Institute of the University of São Paulo School of Medicine’s Clinical Hospital (InCor-HCFMUSP) from 2022 to 2025. The inclusion criteria were: women at postmenopausal period (more than 1 year of amenorrhea); age ≥55 years old; stable chronic CAD (at least one coronary lesion with >50% lumen reduction documented by coronary cineangiography); and body mass index (BMI) ≥ 21.9 kg/m^2^. The exclusion criteria were: dyslipidemia with plasma triglycerides levels ≥500 mg/dL and/or total cholesterol ≥ 300 mg/dL; acute coronary syndrome in the last 6 months prior to the recruitment; congenital heart disease; recent surgery in the last 6 months prior to the recruitment; advanced chronic renal (estimated glomerular filtration rate ≤59 mL/min/1.73 m^2^); liver failure; clinically unstable heart failure or NYHA functional class III or IV; and clinically unstable genetic, hematologic, rheumatic, respiratory, or metabolic diseases. Nutritional consultations at baseline and final periods were scheduled to obtain clinical information, anthropometric measurements, dietary assessment, and physical activity assessment. Also, a written informed consent was obtained at the baseline visit. The subjects were asked to maintain their usual physical activity levels and diet during the study. This study was approved by the Research Ethics Committee (CAAE: 61901722.6.0000.0068) of the University of São Paulo School of Medicine’s Clinical Hospital and approved and registered on the Brazilian Registry of Clinical Trials (RBR-7d7cycj) which can be located at the website https://ensaiosclinicos.gov.br/rg/RBR-7d7cycj. All participants signed informed consent.

### Intervention and outcomes

**Figure 2:**
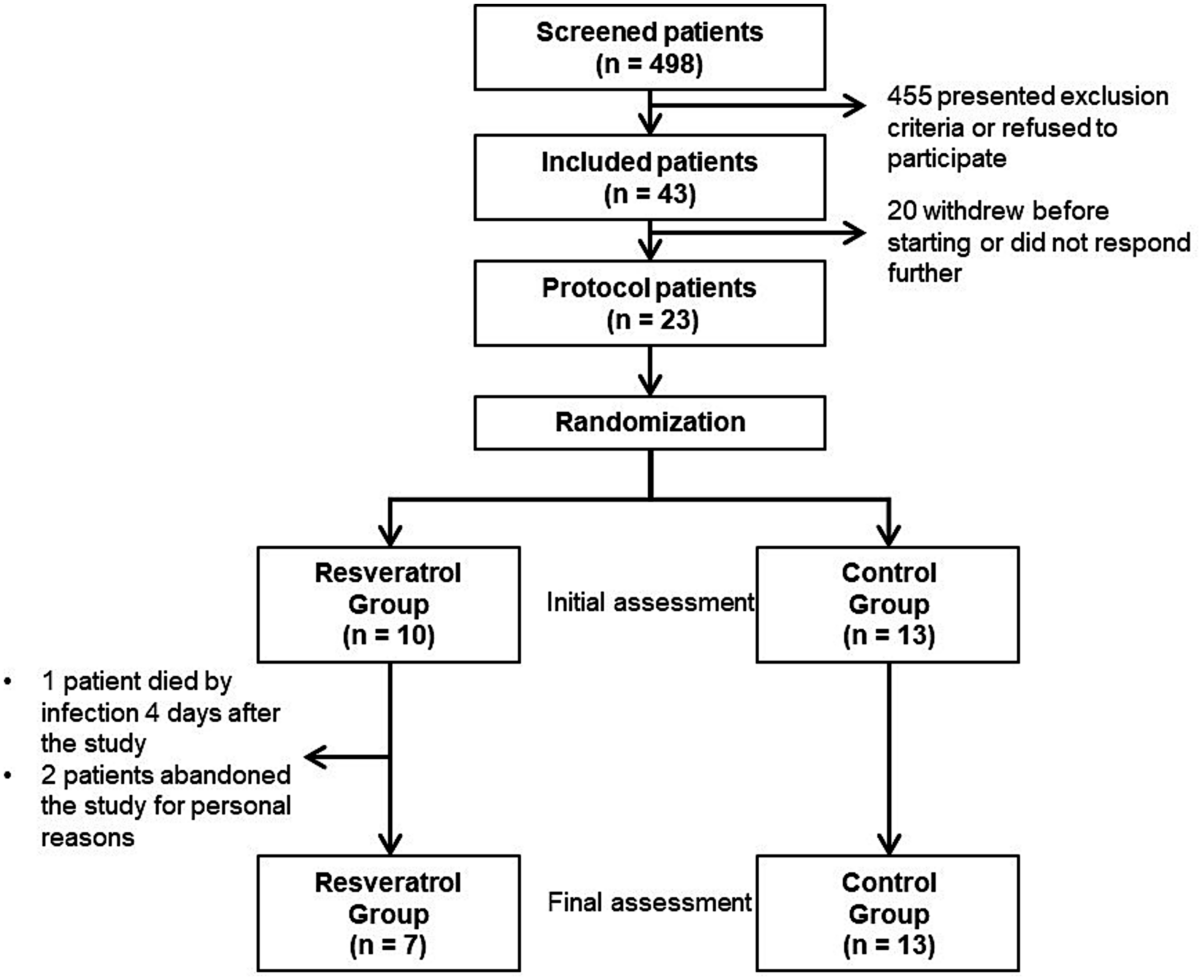
Study’s flow chart.

Participants were randomly assigned to either the intervention or control groups in a 1:1 ratio using random allocation software (SAS) by a researcher who was not involved in direct patient care. Patients were blinded as well as the clinical nutritionist that was directly involved in the patient’s care during the study. The participants in the intervention group received a daily dose of 1,000 mg of resveratrol (2 capsules of 500 mg, which one were taken in the morning and the other in the evening) for 90 days. The resveratrol given consisted of high-purity resveratrol (>98% *trans*-resveratrol) that was obtained from a certified manipulation pharmacy (Formularium, São Paulo, SP, Brazil). The control group received identical capsules with starch (500 mg each capsule) (Figure 2).

The primary outcome of the study was the circulating EPCs (CD34^+^/KDR^+^), and the exploratory outcomes were serum sirtuins 1 and 3 and serum apoptosis biomarkers (caspases 3 and 9, survivin, cIAP2, XIAP, Bcl-2, and cytochrome c).

### Dietary intake, body composition and physical activity measurements

To ensure that the effect found in the study was not due to changes in lifestyle, a dietary assessment of energy, macronutrients, and fibers was performed by applying a 24-h recall in each nutritional consultation and using Brazilian food composition tables (41), as well as assessment of the physical activity levels by applying the short form of the International Physical Activity Questionnaire (IPAQ-Short Form) validated for the Brazilian population (42).

The body composition was assessed by plycometry technique, BMI calculation, and waist circumference. For the percentual body fat estimate, the 4-site skinfold and Durnin and Womersley (43) predictive equations were used.

### Serum and plasma biomarkers

A total of 60 mL of blood was collected after 12 h of fasting and before nutritional consultations. Serum and plasma were separated after 15 min of centrifugation at 1000 *g* at 4 °C of the blood collected in tubes with separating gel and EDTA, respectively. Of the 60 mL of blood collected, 40 mL were collected in heparin tubes for the EPCs analysis in flow cytometry described in the following section. All serum and plasma samples were stored in a −80 °C freezer until the time of analysis.

Serum total cholesterol, triglycerides, HDL-c, and plasma glucose were determined by commercial colorimetric-enzymatic methods (Cholesterol Oxidase Phenol Ampyrone-CHOD-PAP, Merck KGaA, Darmstadtm Germany). LDL-c was calculated using Friedewald equation. Measurements were performed using the automated equipment Dimension RxL (Siemens Healthcare Diagnostics Inc., Newark, DE, USA) with dedicated reagents. The high-sensitivity C-reactive protein (hs-CRP) determined was made by immunonephelometry using dedicated reagents for BN-II equipment from Siemens Healthcare (Marburg, Hessen, Germany). Other serum and plasma biomarkers associated with inflammation and endothelial dysfunction were analyzed using the enzyme-linked immunosorbent assay (ELISA) method, namely: vascular cell adhesion molecule-1 (VCAM-1) (ab223591); calgranulin C (S100A12) (ab282299); interleukin (IL)-6 (ab178013); IL-1β (ab214025); IL-10 (ab185986); tumor necrosis factor-*α* (TNF-α) (ab181421); TNF-α receptor 2 (TNFR2) (ab260061); monocyte chemotactic protein-1 (MCP-1) (ab179886); granulocyte-macrophage colony-stimulating factor (GM-CSF) (ab100529); metalloproteinases-9 (MMP-9) (ab246539); and vascular endothelial growth factor (VEGF) (RK00023). Serum apoptosis biomarkers were also analyzed by ELISA kits, namely: caspase-3 (ab285337); caspase-9 (ab119508); cIAP2 (ab314836); Bcl-2 (ab119506); survivin (ab183361); XIAP (ab155446) e cytochrome c (RK00061). SIRT1 (RK04258) and SIRT3 (ab314347) were also quantified in serum by ELISA KIT. Other markers analyzed that are involved in the mechanisms of action of resveratrol and the study’s outcomes included stem cell factor (SCF) (ab176109), soluble advanced glycation end-products receptor (sRAGE) (ab309111), angiopoietin (Ang)-2 (ab99971), norepinephrine (ab287789), adiponectin (ab314604) and leptin (ab179884). All kits were from Abcam (Cambridge, UK), except for VEGF, cytochrome c, and SIRT1, which were from Abclonal (Massachusetts, USA). A duplicate was perfomed for each analysis.

### Isolation and cryopreservation of PBMCs

Peripheral blood mononuclear cells (PBMCs) were isolated by density-gradient centrifugation using Ficoll–Hypaque (Amersham Bioscience, Sunnyvale, CA, USA). After centrifugation at 800 × *g* for 30 min, the mononuclear cell layer was collected, resuspended in saline solution, and washed twice. Cells were then resuspended in RPMI 1640 medium (Roswell Park Memorial Institute; Sigma, Missouri, USA) and cryopreserved at a concentration of 1 × 10^6^ cells/mL in a freezing solution containing 90% heat-inactivated fetal bovine serum (FBSi; Imunoquímica, Rio de Janeiro, Brazil) and 10% dimethyl sulfoxide (DMSO; Sigma, Missouri, USA). Samples were stored in liquid nitrogen until use.

### Cell thawing and viability assessment

Cryopreserved PBMCs were rapidly thawed in a 37 °C water bath and immediately transferred to tubes containing RPMI 1640 medium supplemented with 10% FBSi. Cell viability was assessed using the Trypan Blue exclusion assay (Merck Millipore, Massachusetts, USA).

### Flow cytometric analysis of endothelial progenitor cells (EPCs)

**Figure 3:**
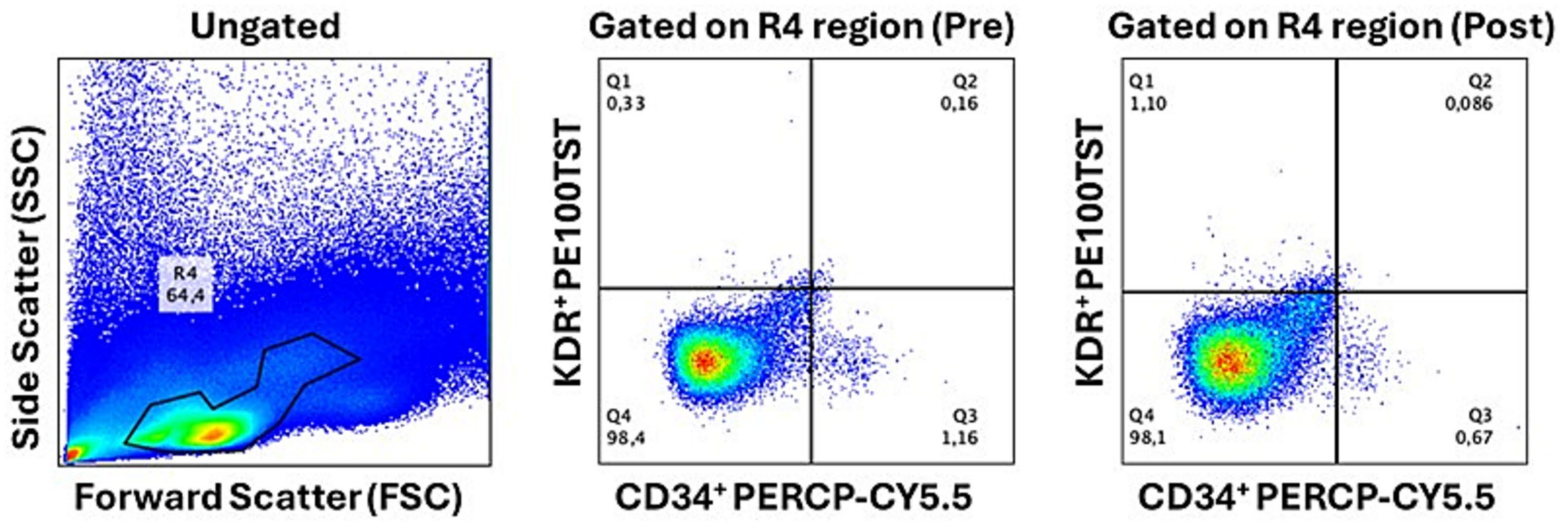
Representative flow cytometry dot plots showing the gating strategy for the characterization of CD34^+^/KDR^+^/CD45^−^ in PBMC.

Endothelial progenitor cells (EPCs) were analyzed by flow cytometry using the following fluorochrome-conjugated antibodies: anti-CD34 PerCP-Cy5.5 (RUO), anti-KDR/VEGFR-2 PE (clone 89), and anti-CD45 FITC (clone 2D1), all obtained from BD Biosciences (California, USA). Samples were analyzed on a FACS Canto II flow cytometer (Becton & Dickinson), within the acquisition of approximately 400,000 events a large, gated selecting CD45-negative cells. Data acquisition and analysis were performed using FlowJo software (version 10.10; Becton Dickinson & Company, CA, USA). EPCs were defined as CD34^+^/KDR^+^ cells within a broad gate encompassing lymphocyte and monocyte populations, restricted to the CD45^−^ cell fraction (Figure 3). Cell viability were also evaluated during the test with the BD Horizon Fixable Viability Stain 450 (FVS 450) kit. The result was expressed as a percentage of CD34^+^/KDR^+^ cells relative to the total number of leukocytes. A duplicate was performed for each analysis.

### Statistical analyses

The sample size was calculated based on a study that evaluated the amount of CD34^+^/KDR^+^ after statin intervention (44). The calculated number of individuals for each of the two groups was five patients, totaling ten patients needed for the study, with a test power of 0.800 using a significance level of *p* < 0.05. The primary outcome (CD34^+^/KDR^+^) was pre-specified and analyzed according to the study protocol. The other biomarker analyses were considered exploratory and hypothesis-generating.

Statistical analyses were conducted using non-parametric tests due to the small sample size. Variables were described according to measures of central tendency and dispersion (median and interquartile range (IQR)) for continuous variables and frequency and percentage for categorical variables. Initially, intragroup differences before and after the intervention were assessed using the paired Wilcoxon test, and the comparison of the group effect was performed using the Mann–Whitney *U* test using the variations (deltas − *Δ*, i.e., post − pre) of the analyzed parameters. Although non-parametric tests were used, standardized mean differences using Hedges’ *g* were calculated for baseline comparisons to provide a scale-independent measure of effect size.

To verify the effect of the intervention on outcomes and associations with covariates, multivariate linear regressions were applied using the forced entry method for the intervention and the stepwise method for the model covariates. The dependent variables were the variations (deltas) of the main variables of the study, such as SIRT1, SIRT3, CD34^+^/KDR^+^, cIAP2, Bcl-2, survivin, XIAP, caspase 9, caspase 3, cytochrome c. As independent variables, in addition to the above variables, age and variations (deltas) in systolic and diastolic blood pressure, heart rate, LDL-c, HDL-c, triglycerides, glucose, hsCRP, body fat percentage, IL-1β, IL-6, IL-10, TNF-α, sTNFR2, S100A12, MMP-9, VEGF, leptin, adiponectin, norepinephrine, GM-CSF, SCF, MCP-1, and angiopoietin 2 were used. The model to be reported in the results was selected using the best adjusted *R*^2^ and without major differences for *R*^2^, highest ANOVA *F* value and with a maximum limit of 4 covariates to avoid overfitting and underfitting of the reported model. *Post hoc* power calculations were conducted to aid interpretation of the results using the G*Power software. Multiple linear regressions with bootstrap resampling with 5,000 iterations were conducted to assess the stability of the estimated effects and bias-corrected and accelerated (BCa) 95% confidence intervals were calculated. Furthermore, multiple linear regression models were performed to adjust for baseline differences in variables showing imbalance between groups.

Subsequently, a mediation analysis was applied using the Baron and Kenny method (45) to verify whether the variables in the regression models described above act as mediators of the effect of resveratrol on the study’s outcome variables. The direct effect of resveratrol (*c’*) is the effect of resveratrol on the outcome (i.e., *B* coefficient) obtained in a multivariate linear regression with forced entry method using the covariates of the models reported above. The effect of resveratrol on the possible mediating variables (*a*) is the *B* coefficient of a simple linear regression of each variable of the model (if there was more than one possible mediator, then the model has *a1*, *a2*, etc.). The effect of possible mediating variables on the dependent variable (*b*) is the *B* coefficient of the mediators obtained in the multiple regression. The associations are represented below:


$$\text{Indirect effect}=X\overset{a}{\to }M\overset{b}{\to }Y$$

The indirect effect of resveratrol was calculated by a*b, and Sobel’s test was applied to assess statistical significance. For all analyses, the two-tailed significance level considered was *p* < 0.05. The statistical analysis was carried out in SPSS software version 20.0.

## Results

Most patients presented triple-vessel CHD and comorbidities in both groups (Table 1). Overall diet and physical activity were maintained throughout the study (Supplementary Table 1). Baseline characteristics did not differ among groups, apart from HDL-c (*p* = 0.014; data not shown) and serum S100A12 (*p* = 0.024; data not shown), which showed moderate to large differences between groups (Hedges’ *g* = 1.52 for HDL-c and *g* = 0.90 for S100A12) (Table 2).

**Table 1:** Clinical characteristics of the participants.

Variables	Placebo (*n* = 13)		Resveratrol (*n* = 7)	
	Frequency	%	Frequency	%
Clinical characteristics				
*CHD classification*				
Single-vessel	1	7.7	0	0.0
Double-vessel	1	7.7	0	0.0
Triple-vessel	11	84.6	7	100.0
*Alcohol consumption*	1	7.7	1	14.3
*Smoking status*				
Smoker	3	23.1	2	28.6
Former smoker	2	15.4	2	42.9
*Comorbidities*				
Chronic kidney disease	1	7.7	1	14.3
Rheumatoid arthritis	1	7.7	0	0.0
Type 1 diabetes mellitus	1	7.7	0	0.0
Type 2 diabetes mellitus	3	23.1	2	28.6
Heart failure	1	7.7	2	28.6
Systemic lupus erythematosus	0	0.0	1	14.3
Stroke	1	7.7	1	14.3
Medications				
*Calcium channel blockers*				
Amlodipine	8	61.5	6	85.7
*Antiplatelet agents*				
ASA	12	92.3	7	100.0
Clopidogrel	13	100.0	7	100.0
*Beta-blockers*				
Carvedilol	3	23.1	1	14.3
Atenolol	9	69.2	5	71.4
Metoprolol	1	7.7	0	0.0
*ACE inhibitors*				
Enalapril	3	23.1	3	42.9
*ARB*				
Losartan	9	69.2	3	42.9
*Statins*				
Atorvastatin	13	100.0	7	100.0
*Diuretics*				
Hydrochlorothiazide	4	30.8	2	28.6
Chlorthalidone	1	7.7	0	0.0
Furosemide	1	7.7	1	14.3
Spironolactone	2	15.4	0	0.0
*Hypoglycaemic agents*				
Metformin	6	46.2	5	71.4
Glicazide	5	38.5	5	71.4
Insulin	3	23.1	0	0.0
Dapagliflozin	2	15.4	3	42.9
*Vasodilators*				
Propranolol	1	7.7	1	14.3
Isosorbide dinitrate	4	30.8	1	14.3
Isosorbide mononitrate	3	23.1	1	14.3
*Antilipemic*				
Ezetimibe	2	15.4	0	0.0
*Alpha-1-adrenergic agonists*				
Clonidine	1	7.7	0	0.0
*Proton pump inhibitors*				
Omeprazole	9	69.2	4	57.1
*Other medications*				
Methotrexate	1	7.7	0	0.0
Colchicine	1	7.7	0	0.0
Bromazepam	1	7.7	0	0.0
Amitriptyline	1	7.7	0	0.0
Sertraline	1	7.7	0	0.0
Domperidone	1	7.7	0	0.0
Gabapentin	1	7.7	0	0.0
Paroxetine	1	7.7	0	0.0

**Table 2:** Effects of resveratrol on circulating sirtuins, EPCs, and apoptosis biomarkers.

Models (variable’s *Δ*)	Adjusted *R*^2^	*B*	*SE*	*p*	95% CI	
					Lower	Higher
Sirtuin 1, ng/mL	0.739					
Resveratrol		0.00200	0.00300	0.395	−0.003	0.008
Norepinephrine, pg/mL		0.00002	0.00000	**0.000**	**0.000**	**0.000**
Survivin, pg/mL		0.00002	0.00000	**0.001**	**0.000**	**0.000**
Sirtuin 3, ng/mL	0.786					
Resveratrol		−0.557	0.375	0.158	−1.355	0.242
XIAP, ng/mL		−0.143	0.020	**0.000**	**−0.185**	**−0.101**
Adiponectin, μg/mL		−0.388	0.103	**0.002**	**−0.607**	**−0.169**
CD34^+^/KDR^+^, %	0.605					
Resveratrol		−0.200	0.048	**0.001**	**−0.304**	**−0.096**
sTNFR2, ng/mL		0.060	0.033	0.088	−0.010	0.130
HDL-c, mg/dL		−0.026	0.007	**0.002**	**−0.040**	**−0.012**
XIAP, ng/mL		−0.009	0.003	**0.002**	**−0.015**	**−0.004**
cIAP2, ng/mL	0.371					
Resveratrol		−0.042	0.176	0.816	−0.414	0.331
Bcl-2, ng/mL		0.216	0.063	**0.003**	**0.083**	**0.348**
Bcl-2, ng/mL	0.746					
Resveratrol		−0.004	0.367	0.991	−0.786	0.778
S100A12, pg/mL		0.001	0.000	**0.001**	**0.000**	**0.001**
cIAP2, ng/mL		1.549	0.403	**0.002**	**0.689**	**2.409**
MCP-1, pg/mL		0.014	0.005	**0.012**	**0.003**	**0.024**
Survivin, pg/mL	0.834					
Resveratrol		−34.575	53.403	0.527	−148.401	79.251
IL-1β, pg/mL		−4.815	0.528	**0.000**	**−5.939**	**−3.690**
Leptin, ng/mL		−3.270	0.986	**0.005**	**−5.372**	**−1.168**
Angiopoietin 2, pg/mL		−0.659	0.223	**0.010**	**−1.135**	**−0.183**
XIAP, ng/mL	0.818					
Resveratrol		−3.968	1.888	0.053	−7.992	0.055
Sirtuin 3, ng/mL		−5.476	0.641	**0.000**	**−6.842**	**−4.110**
Adiponectin, μg/mL		−1.880	0.599	**0.007**	**−3.157**	**−0.603**
GM-CSF, pg/mL		2.081	0.879	**0.032**	**0.207**	**3.956**
Caspase 9, ng/mL	0.787					
Resveratrol		−7.152	3.003	**0.031**	**−13.554**	**−0.751**
hsCRP, mg/L		8.320	1.007	**0.000**	**6.175**	**10.465**
TNF-α, pg/mL		0.321	0.052	**0.000**	**0.211**	**0.432**
Age, years		1.033	0.255	**0.001**	**0.489**	**1.577**
Caspase 3, ng/mL	0.021					
Resveratrol		−1.225	1.031	0.250	−3.390	0.941
Cytochrome c, pg/mL	0.424					
Resveratrol		18.913	18.663	0.326	−20.650	58.477
Body fat, %		14.789	4.497	**0.005**	**5.256**	**24.321**
Sirtuin 1, ng/mL		−2041.741	902.627	**0.038**	**−3955.224**	**−128.257**

Values in bold indicate statistical significance.

At the end of the study, it was observed an increase in cardiac frequency was observed (*p* = 0.027) as well as in the serum apoptosis inhibitor proteins cIAP2 (*p* = 0.018) and Bcl-2 (*p* = 0.043) in the resveratrol group. Although there has been no statistically significant reduction in XIAP (*p* = 0.068) in the resveratrol group, a significant difference in variations (*Δ*) among groups was detected in this marker (*p* = 0.047), indicating that the resveratrol intervention showed a trend towards a reduction in serum XIAP levels compared with placebo group. Serum VEGF levels variations (*Δ*) also were significantly higher in resveratrol group (*p* = 0.029), however, most of the measurements of this marker were below the detection limit, hindering the interpretation of the actual effect of resveratrol on this marker (Supplementary Table 4).

Further associations assessed the effect size of resveratrol and covariates on the outcomes through multiple linear regression analysis (Table 2). The present study did not show significant effect of resveratrol on serum SIRT1 (*p* = 0.395) and SIRT3 (*p* = 0.158). However, survivin (*p* = 0.001) and norepinephrine were significant predictors of SIRT1, just as XIAP (*p* < 0.001) and adiponectin (*p* = 0.002) were significant predictors of SIRT3. Regarding apoptosis biomarkers, resveratrol was shown to decrease serum caspase 9 (*p* = 0.031) when adjusted for C-reactive protein (*p* < 0.001), TNF-*α* (*p* < 0.001), and age (*p* = 0.001), while there was no significant effects of resveratrol on the other biomarkers. As for the circulating CD34^+^/KDR^+^ cells, resveratrol initially has been shown to have a significant association with reduction (*B* = −0.249; SE = 0.054; *p* < 0.001; 95% CI = −0.364 to −0.133) when adjusted for sTNFR2 (*B* = 0.143; SE = 0.004; *p* < 0.001; 95% CI = 0.135–0.151), HDL-c (*B* = −0.030; SE = 0.008; *p* < 0.002; 95% CI = −0.046 to −0.013), and XIAP (*B* = −0.011; SE = 0.003; *p* < 0.002; 95% CI = −0.017 to −0.005), with the model explaining 99.4% (adjusted *R*^2^ = 0.994) of the variation in circulating EPC (data not shown). Within this linear regression model, it was found that sTNFR2 explained 98.1% of the circulating EPC variation (*B* = 0.137; SE = 0.004; *p* < 0.001; 95% CI = 0.128–0.147; adjusted *R*^2^ = 0.981) in a simple linear regression (data not shown). An influential observation was identified, associated with an extreme increase in sTNFR2 in the placebo group. Exclusion of this observation for a sensitivity analysis substantially reduced the adjusted *R*^2^ (from 0.994 to 0.605), indicating that the model is highly sensitive to this observation, and showed that resveratrol intervention remained significantly independently associated (*p* = 0.001) with circulating EPC when adjusted for HDL-c (*p* = 0.002), XIAP (*p* = 0.002), and sTNFR2 (*p* = 0.088), with the latter losing the association following the removal of the outlier observation (Table 2). No other anomalous associations indicating further outliers or exhibiting an extremely high adjusted *R*^2^ were found in the other regression models.

Furthermore, overall size effect and power of the multiple linear regression models were large, including the SIRT1 (*f*^2^ = 2.83; power = 0.999), SIRT3 (*f*^2^ = 3.67; power = 0.999), CD34^+^/KDR^+^ (*f*^2^ = 1.53; power = 0.978), Bcl-2 (*f*^2^ = 2.937; power = 0.999), survivin (*f*^2^ = 5.024; power = 1.000), XIAP (*f*^2^ = 4.495; power = 0.999), and caspase 9 (*f*^2^ = 3.695; power = 0.999) models. The underpowered models included cIAP2 (*f*^2^ = 0.590; power = 0.640), caspase 3 (*f*^2^ = 0.022; power = 0.094), and cytochrome c (*f*^2^ = 0.736; power = 0.753) models. Given the high power of most regression analyses, the stability of the variables analyzed by multiple linear regression with bootstrapping method showed the robustness of the previous results (Table 3). The effect of resveratrol on circulating CD34^+^/KDR^+^ cells has shown to be robust and stable (BCa 95% CI = −0.320 to −0.084), as well as the predictors sTNFR2 (BCa 95% CI = 0.002–0.144) and HDL-c (BCa 95% CI = −0.040 to −0.011), whereas XIAP was unstable (BCa 95% CI = −0.038 to 0.037). However, the effect of resveratrol on caspase 9, which was previously statistically significant and large, has shown to be unstable (BCa 95% CI = −10.723 to 3.085). No stability of the effects of resveratrol was found in the other biomarkers (Table 3). Moreover, no statistically significant mediation effects were observed in any outcome (Supplementary Table 2). When adjusted by baseline HDL-c and S100A12, which differed among groups, the effects of resveratrol were not statistically significant for any of the biomarkers (Supplementary Table 3). However, baseline was a statistically significant predictor of SIRT3 (*p* = 0.033), XIAP (*p* = 0.029), and cytochrome c (*p* = 0.025) (Supplementary Table 3).

**Table 3:** Multiple linear regression with bootstrap analysis.

Models (variable’s Δ)	*B*	Bias	*SE*	BCa 95% CI	
				Lower	Higher
Sirtuin 1, ng/mL					
Resveratrol	0.00200	0.00000	0.00200	−0.00300	0.00900
Norepinephrine, pg/mL	0.00002	0.00000	0.00001	**0.00000**	**0.00004**
Survivin, pg/mL	0.00002	−0.00001	0.00002	−0.00001	0.00002
Sirtuin 3, ng/mL					
Resveratrol	−0.557	0.063	0.349	−1.346	0.411
XIAP, ng/mL	−0.143	0.000	0.057	**−0.241**	**−0.006**
Adiponectin, μg/mL	−0.388	0.030	0.164	−0.722	0.025
CD34^+^/KDR^+^, %					
Resveratrol	−0.200	0.002	0.052	**−0.320**	**−0.084**
sTNFR2, ng/mL	0.060	0.003	0.033	**0.002**	**0.144**
HDL-c, mg/dL	−0.026	0.000	0.007	**−0.040**	**−0.011**
XIAP, ng/mL	−0.009	0.002	0.006	−0.038	0.037
cIAP2, ng/mL					
Resveratrol	−0.042	0.015	0.150	−0.465	0.328
Bcl-2, ng/mL	0.216	0.006	0.094	**0.025**	**0.402**
Bcl-2, ng/mL					
Resveratrol	−0.004	0.046	0.388	−0.861	0.974
S100A12, pg/mL	0.001	0.000	0.000	**0.000**	**0.001**
cIAP2, ng/mL	1.549	0.026	0.562	**0.280**	**3.153**
MCP-1, pg/mL	0.014	−0.001	0.006	**0.001**	**0.024**
Survivin, pg/mL					
Resveratrol	−34.575	24.333	52.085	−225.159	202.449
IL-1β, pg/mL	−4.815	1.651	2.665	−5.948	1.184
Leptin, ng/mL	−3.270	1.544	1.988	N/A^a^	N/A^a^
Angiopoietin 2, pg/mL	−0.659	0.354	0.472	−2.144	0.796
XIAP, ng/mL					
Resveratrol	−3.968	0.105	2.160	−7.918	0.455
Sirtuin 3, ng/mL	−5.476	0.943	2.281	−8.381	7.164
Adiponectin, μg/mL	−1.880	−0.013	1.030	−3.497	0.089
GM-CSF, pg/mL	2.081	−1.000	1.590	−0.993	2.667
Caspase 9, ng/mL					
Resveratrol	−7.152	1.561	3.522	−10.723	3.085
hsCRP, mg/L	8.320	−1.296	3.162	−0.340	9.979
TNF-α, pg/mL	0.321	−0.064	0.126	**0.040**	**0.371**
age, years	1.033	−0.230	0.496	**0.093**	**1.347**
Caspase 3, ng/mL					
Resveratrol	−1.225	0.000	0.953	−3.260	0.846
Cytochrome c, pg/mL					
Resveratrol	18.913	−1.229	17.724	−13.342	49.454
Body fat, %	14.789	−1.047	6.289	**1.573**	**23.768**
Sirtuin 1, ng/mL	−2041.741	264.330	4749.463	−3358.103	675.813

a Bootstrap BCa confidence interval could not be computed. Values in bold indicate statistical significance.

## Discussion

As far as it is known, this is the first study that assessed the effects of resveratrol on EPCs and multiple circulating apoptosis biomarkers for CHD treatment of postmenopausal women in a clinical research setting. The main findings of the study are that resveratrol modulated serum anti-apoptotic markers and reduced the EPCs count in postmenopausal women with CHD.

### Apoptosis biomarkers

In advanced stages of atherosclerosis, inflammation and apoptosis of endothelial cells and VSMCs contribute to the increased plaque rupture risk (14). It was previously shown that the fibrous cap and necrotic core from carotid arteries of symptomatic patient plaques had higher expression of caspase 3 and lower VSMCs compared to asymptomatic patients, suggesting that increased apoptosis leads to increased severity of atherosclerosis (26). Furthermore, results of the same study showed that plaques from symptomatic patients had higher expression of cIAP2, XIAP and survivin, suggesting a defense mechanism to stabilize plaque and prevent acute coronary events, since these proteins inhibit the activity of caspases 3, 7, and 9 (26). Animal model studies showed that the overexpression of the anti-apoptotic proteins cIAP2 and Bcl-2 protected the heart tissue against I/R injury and inhibited cardiac apoptosis, leading to a reduced infarct size and better recovery after the coronary artery occlusion (46, 47). Moreover, apoptosis induced by myocardial infarction is characterized by reduction of Bcl-2 expression and augmentation of Bax expression, demonstrating the significance of Bcl-2 in cardiac lesion protection (12). Endothelial homeostasis is also affected by IAPs and Bcl-2. It has been shown that endothelial cells undergoing hypoxia express cIAP2 due to the stress, preventing apoptosis, and that increasing its expression through an agonist leads to decrease in caspase 3 activity and increased endothelial cell survival (48, 49). Bcl-2, on the other hand, has an anti-apoptotic effect on endothelial cells due to its mechanisms of inhibiting the release of cytochrome c, increasing survivin expression, and its antioxidant effects (50–53). XIAP and survivin also have been implicated in inhibition of apoptosis in endothelial cells, indicating a potential vascular protective effect (52, 54–56).

Initial findings of the present study showed that resveratrol treatment in postmenopausal woman with CHD increased serum concentrations of two apoptosis inhibition proteins, cIAP2 and Bcl-2, which could be protective to endothelial cells. In addition, these two circulating proteins were associated with each other in multivariate analysis, suggesting a close physiological connection. Other findings of the present study showed that MCP-1 and S100A12, markers of endothelial dysfunction and cell damage respectively, were significant predictors of Bcl-2 increase, indicating that the rise in the anti-apoptotic levels could be a defense mechanism contributing to maintaining endothelial homeostasis (50–53). It is also important to note that resveratrol treatment was associated with reduction in serum caspase 9 when adjusted for age and inflammatory biomarkers (hsCRP and TNF-α), suggesting, alongside increase of cIAP2 and Bcl-2 levels, an anti-apoptotic effect. Although hsCRP and TNF-α were significant predictors of serum caspase-9, no significant effect of resveratrol on these biomarkers was found. This implies that the inflammatory response increases signaling in the intrinsic apoptosis pathway, and that high-dose resveratrol supplementation modulate this pathway in women with CHD, which could be protective for endothelial cells. This hypothesis is supported by previous laboratory studies showed that resveratrol reduced endothelial cell apoptosis (57–62). By decreasing apoptosis signaling, resveratrol has been reported to reduce I/R damage in cardiac tissue and decrease the formation of apoptotic debris, facilitating and increasing efferocytosis (63). Despite the findings of the current study, there is a lack of clinical studies evaluating the effects of resveratrol on circulating markers of apoptosis in literature, and one of the few studies showed that resveratrol reduced cytokeratin-18 M30, a marker of hepatocellular apoptosis, in non-alcoholic fatty liver disease (NAFLD) patients, which was associated with reduction of hepatic tissue echogenicity as well, suggesting a better clinical outcome (64). Other studies showed that increased circulating apoptosis biomarkers such as Fas and Fas-ligand (FasL) were associated with cardiometabolic risk factor clusters (17), impaired left ventricular strain (19), AMI and angina pectoris (18), and increased cardiovascular risk (16), indicating that reduction of pro-apoptotic biomarkers or increase of anti-apoptotic biomarkers could be associated with reduced risk. On the other hand, the current study did not evaluate circulating Fas, FasL nor cytokeratin-18 M30. Although the literature suggests that these biomarkers influence the pathogenesis and risk of CVD, no studies have assessed their impact on clinical outcomes, making it difficult to establish a causal link. Furthermore, the present study did not assess clinical outcomes either, so interpretations should be made with caution.

The mechanisms by which resveratrol inhibits apoptosis is through activation of AMPK/SIRT1/PGC-1α pathway (57), enhancing superoxide dismutase (SOD2) activities and glutathione (GSH) content (58, 60, 65), regulation of mitochondrial ROS levels by SIRT3 activation (60), and inhibition of NF-κB activation, NAPDH oxidase activity and consequent inhibition of ROS production (62). However, findings of the present report showed lack of effects of resveratrol on circulating SIRT1 and SIRT3 and did not assess oxidative stress markers. Furthermore, studies showed that elevation of sirtuins levels occurs alongside with decline in SIRT activity in situations of chronic inflammation and oxidative stress, such as non-communicable diseases (66). Nevertheless, the present study did not assess sirtuins activity, and circulating sirtuins may not reflect the effects of resveratrol on intracellular sirtuins. In the present study, the effects of resveratrol on circulating sirtuins also were shown to be unstable, despite the large effect size and power. This indicates a high degree of uncertainty that can be attributed to the small sample of the study.

Although resveratrol has previously been shown to inhibit caspase 3 (65), no significant effect on circulating levels was observed in the current study. Moreover, no significant effect was observed in circulating cytochrome c, a reported marker of mitochondrial outer membrane permeabilization which is triggered by the intrinsic apoptosis pathway (67). The effector caspase 3 is activated by the apoptosome, which is a complex formed by cytochrome c, apoptotic protease-activating factor 1 (APAF-1), and the initiator caspase 9 can affect the entire process described (67). The present study found a reduction in caspase 9 after multiple adjustments, suggesting inhibition of intrinsic apoptosis. Reduction of serum caspase 9 with anakinra treatment was associated with improvement of left ventricular function in rheumatoid arthritis patients (19). Moreover, it was previously shown that atherosclerotic human arteries expresses caspases 9 and 3 in endothelial cells and fibrous plaques, along with reduced expression of Bcl-2, indicating involvement of mitochondrial dysfunction in atherosclerotic apoptosis (68). Other study showed that the treatment of human coronary endothelial cells with oxidized LDL (ox-LDL) led to Bcl-2 and cIAP expression reduction, as well as caspases 9 and 3 activation, resulting in increased apoptosis (69). However, other study using human aorta showed that in the course of the progression of atherosclerosis, the expression of caspase 3 was found reduced whereas the expression of caspase 9 was increased (70). The authors argue that the fall in caspase 3 found was due to increased cellular senescence and predominance of necrotic processes in atherosclerotic plaques (70). In myocardial tissue of individuals with severe heart failure and left ventricular assist devices, various pro-inflammatory cytokines and caspase 9 were found increased, whilst the expression of caspase 3 was found to be reduced (71). Although plasma caspase 3 was associated with coronary calcium (CAC), abdominal aortic wall thickness, and aortic compliance in epidemiological setting (72), a cohort showed that it was not associated with coronary events (73). The fact that some studies showed that caspase-3 levels are already reduced in cardiovascular diseases may explain why the present study found no effect of resveratrol on serum levels. However, it is difficult to draw conclusions regarding the effect of caspase 9, Bcl-2, and cIAP2 modulation observed in this study on apoptosis, given that the use of circulating markers may be non-specific for apoptotic signaling pathways in cardiac and vascular tissues. Furthermore, the associations of resveratrol treatment and caspase 3 and cytochrome c were underpowered and unstable after bootstrapping. It is important to note that Bcl-2 and cIAP2 modulation lost significance after multiple adjustments, and that caspase 9 associations were also unstable after bootstrapping, indicating that although the associations were significant, they were not robust in the resampling, and the effect should be interpreted with caution.

Additionally, there was no significant effect of resveratrol on circulating survivin. Another finding was trend towards XIAP reduction in the resveratrol group and significant differences of XIAP variations between groups. Data from the literature suggests an interaction between IAPs, as a compensatory mechanism of cIAP2 upregulation was found in XIAP-deficient mice (74), which could explain the XIAP reduction concurrently with a cIAP2 increase found in the present study. Furthermore, the pharmacological affinity of resveratrol for IAPs may vary, suggesting a greater selectivity for cIAP2 and Bcl-2 (75). Nevertheless, bootstrapping analyses revealed lack of robustness in the IAP analyses, requiring cautious interpretation.

### Circulating endothelial progenitor cells

Modulation of endothelial cell apoptosis may affect key cells of endothelial homeostasis, such as EPCs. After statistical adjustments, the current study showed that resveratrol intervention reduced significantly the EPCs levels, even after exclusion of outliers, as mentioned in the results section. Also, this result remained stable after bootstrapping analysis, indicating robust results with strong contributions of resveratrol to the variation of EPCs. This was an unexpected result, since higher circulating EPCs have been associated with better endothelial function (28), and data in the literature suggests that resveratrol improves endothelial and vascular functions (76–80). Nevertheless, it is relevant to consider diverse EPCs phenotypes for interpretation. When first isolated from PBMC by Asahara *et al*., EPCs were identified as CD34^+^/KDR^+^ and described as circulant immature cells relevant in postnatal angiogenesis or vascular repair through circulating EPCs recruitment and subsequent re-endothelization (81), which were the same markers used in the current study. However, it has been discussed whether circulating or local tissue EPCs are the major participants of vascular regeneration, and which surface proteins should be used to classify EPCs (82). Nevertheless, higher numbers of circulating EPCs has been associated with better endothelial function and lower cardiovascular risk (28–31). Furthermore, the cardiovascular protection found in women in the fertile period has been attributed to estrogen’s effects on EPCs recruitment and function, including increasing proliferation, angiogenesis, and NO secretion (32, 33). Postmenopausal women with CHD also presented lower levels of EPCs than healthy postmenopausal women, and the latter group had higher levels than age-matched healthy men, highlighting different influences of sex and menopausal state on EPCs levels and the negative effects the biomarker caused by CHD progression (34). Conversely, it was shown that EPCs levels rise sharply after acute myocardial infarction (AMI), although they do not reach the same levels as in healthy individuals (83). Another study showed that circulating EPCs were highest in individuals with CHD selected for revascularization according to disease severity (84). As previously mentioned, EPCs count increase with disease severity possibly as a repair mechanism triggered by endothelial damage. Thus, considering that in the current study most of the participants had triple vessel CHD, it is possible that a reduction in endothelial dysfunction and damage causes a decrease in EPCs signaling, leading to a reduced circulation count.

Considering the reported findings on apoptosis biomarkers, an increase in EPCs count was expected due to the supposed apoptosis inhibition indicated by caspase 9 decrease along with and increase in cIAP2 and Bcl-2. However, other interpretations are also possible. Apoptotic bodies of endothelial cells and alarmins secreted from dead cells induce proliferation, mobilization and homing of EPCs, and inhibition of apoptosis in mature endothelial cells could decrease mobilization signaling of EPCs, consequently reducing their circulating count, as previously reported (85, 86). Despite that, in the present report, the circulating levels of S100A12 and their soluble receptor sRAGE, respectively an alarmin and a soluble receptor, did not change with resveratrol intervention. An alternative explanation for the reduction in circulating EPCs following resveratrol treatment is the occurrence of increased homing for endothelial repair, as the polyphenol has been shown to increase EPCs proliferation, adhesion, and migration in a dose-dependent manner (87). The hypothesis that the EPCs count change due to mobilization to damaged tissues was also suggested in a study showing that patients with chronic obstructive pulmonary disease (COPD) and endothelial dysfunction reduced circulating EPCs after exercise treatment as well as improvement in endothelial function, marked by increased reactive hyperemia index (88). Another study showed that hysterectomy in female rats resulted in an increase in circulating EPCs, suggesting that removal of the target organ prevented circulating EPCs from being recruited to sites of endometrial regeneration/remodeling, thereby increasing their circulating count (33). It is important to note that endothelial function using a standard technique such as flow-mediated dilation (FMD) was not assessed in the present study, limiting effects on endothelial function. Additionally, circulating markers of inflammation, which are associated with endothelial dysfunction, were not modulated by resveratrol. Moreover, since evidence suggest that increased circulating EPCs levels are associated with cardiovascular protection (28–31), it is difficult to draw conclusions regarding the hypothesis that the reduction of EPCs was due to increased mobilization and recruitment, as the resveratrol intervention showed no effects on EPCs mobilizing factors such as angiopoietin 2 and stem cell factor (SCF). One factor that is present in cardiovascular diseases and may underlie this lack of effect is bone marrow endothelial dysfunction, which decreases angiogenesis and impairs vascular regeneration (89).

### Strengths and limitations

This study has limitations that must be considered for a proper interpretation. First, only circulating metabolic and apoptosis biomarkers were used, which makes it difficult to understand the intracellular and cardiovascular-specific effects of the intervention. However, this approach reflects systemic changes rather than changes in specific cells and has potential for clinical use. Previous studies using resveratrol and other interventions types showed modulation of circulating biomarkers such as SIRT1 (80, 90–92). Despite that, circulating sirtuins do not reflect sirtuins activity in the cell, which is limiting. The present study also did not assess FMD to reflect the endothelial function due to impracticability and high cost of the project but circulating biomarkers of endothelial function were measured, limiting interpretations regarding endothelial function. On the other hand, circulating biomarkers of inflammation and endothelial dysfunction were assessed, such as VCAM-1, MCP-1, TNF-α. However, no significant effects were detected. It is also important to note that the biomarker analyses were exploratory and hypothesis-generating, and given the large number of comparisons, results should be interpreted with caution. It was found that most of the measurements of VEGF were below the detection limit, suggesting a limitation in analytical sensitivity for this specific population. To address this issue, this marker was not used in multivariate analyses.

Another consideration is that baseline HLD-c and S100A12 differed among groups, and after adjustment for these variables, no significant effects of resveratrol were detected. Nevertheless, the main multiple regression analyses used the stepwise method to identify the variables that contributed most to the variation in the study outcomes, which could be examined in future studies. Furthermore, the mediation analysis showed no significant associations. The absence of a significant indirect effect may be related to the limited sample size and power, as mediation analyses require greater statistical power to detect indirect effects. Although the analysis had sufficient statistical power for multivariate analysis, a lack of robustness and stability was detected using the bootstrap method. This may be attributed to the small sample and the high variability of the biomarkers studied.

Other important points to consider relate to EPCs. First, there is much debate surrounding the EPCs definition. The main concept behind EPCs is that they are cells capable of adhering to the endothelium, a property that is assessed using an *in vitro* method (93). It is worth highlighting that “early” EPCs and “late” EPCs, distinguished by *in vitro* methods, exhibit different abilities to adhere to the endothelium, and the CD34^+^/KDR^+^ phenotype may not differentiate these EPCs. Early EPCs exert a greater angiogenic effect through the paracrine secretion of growth factors than through reendothelialization (93). On the other hand, regardless of the degree of maturation of the EPCs, both contribute to endothelial regeneration, and the present study used a phenotype that is supported by epidemiological data which show associations with clinical outcomes (28–31). Additionaly, the gating strategy used in the present study covered areas of lymphocytes and monocytes because peripheral blood EPCs are found in both regions (94, 95). Since peripheral blood EPCs are associated with cardiovascular protection, the reduction found after resveratrol treatment in the present study may also indicate an increased risk. It was also found an increase in heart rate in the resveratrol group, which could be associated with increased risk (96). Future studies should investigate the long-term clinical impact of high dose of resveratrol in chronic CHD patients with triple-vessel disease, as the present study has a short follow-up period and a small sample, which may have affected the internal validity and the interpretation of the effects on cardiovascular risk factors and inflammatory markers. Furthermore, circulating resveratrol was not assessed in the present study, limiting the interpretation of bioavailability of resveratrol.

Regarding the strengths of the study, several circulating biomarkers related to inflammation, apoptosis, endothelial dysfunction and mechanisms of action of resveratrol were analyzed in the present study. The study includes most multivariate statistical analyses with statistical power, despite the small sample. Furthermore, robustness of associations was assessed by bootstrapping techniques, which showed a degree of uncertainty in apoptosis biomarkers despite the statistical significance, but a robust and stable effect of resveratrol on circulating EPCs, which should be replicated in future studies, as external validity of these results is required. Other strengths include the high adherence to the treatment (> 85%) and the use of commercial kits for assessing the circulating biomarkers, which are easily replicated. Future studies should assess how EPCs behave in CHD together with FMD and oxidative stress.

## Conclusion

The main results of the study showed that resveratrol increased serum Bcl-2 and cIAP2, and also reduced caspase 9 and circulating EPCs in postmenopausal women with chronic CHD, likely unrelated to circulating SIRT1 and SIRT3. The effects of resveratrol treatment in this population group should be assessed with caution, as a reduction in EPCs is associated with impaired endothelial function and an increased risk of cardiovascular disease.

## Data Availability

The raw data supporting the conclusions of this article will be made available by the authors, without undue reservation.
