# Supplementary material for: The effects of resveratrol on endothelial progenitor cells and apoptosis biomarkers in postmenopausal women with chronic coronary heart disease: a randomized controlled trial
Source: Front Nutr. 2026 May 14;13:1814668. doi: 10.3389/fnut.2026.1814668 (PMC13215931; doi:10.3389/fnut.2026.1814668)
Supplement: Supplementary file 1 [file Data_Sheet_1.zip › Supplementary Table 4.docx]

**Supplementary Table 4.** Characteristics of the participants at baseline and post-treatment.

| **Variables** | **Placebo (n = 13)** | | | | | |  | **Resveratrol (n = 7)** | | | | | | |  | |  | |
| --- | --- | --- | --- | --- | --- | --- | --- | --- | --- | --- | --- | --- | --- | --- | --- | --- | --- | --- |
|  | **Baseline** | | | **Post-intervention** | | |  | **Baseline** | | | **Post-intervention** | | | |  | | **Δ** | |
|  | **Median** | **IQR** | | **Median** | **IQR** | | ***p*** | **Median** | **IQR** | | **Median** | **IQR** | | ***p*** | | ***p*** | |  |
| Age, *years* | 69.0 | 62.0 | 71.0 | N/A | N/A | | N/A | 72.0 | 70.0 | 75.0 | N/A | N/A | | N/A | | N/A | |  |
| ***Risk factors*** |  |  |  |  |  |  |  |  |  |  |  |  |  |  | |  | |  |
| Systolic BP, *mmHg* | 136.0 | 121.5 | 142.0 | 132.0 | 120.0 | 153.0 | 0.609 | 130.0 | 126.0 | 142.0 | 124.0 | 117.0 | 140.0 | 0.310 | | 0.088 | |  |
| Diastolic BP, *mmHg* | 73.0 | 69.0 | 82.5 | 76.0 | 66.5 | 83.5 | 0.929 | 75.0 | 68.0 | 78.0 | 68.0 | 50.0 | 80.0 | 0.075 | | 0.103 | |  |
| Heart rate, *bpm* | 64.0 | 60.5 | 70.5 | 64.0 | 60.5 | 70.5 | 0.824 | 67.0 | 51.0 | 73.0 | 72.0 | 60.0 | 78.0 | **0.027** | | 0.051 | |  |
| Total cholesterol, *mg/dL* | 149.0 | 130.0 | 200.0 | 156.0 | 139.0 | 195.5 | 0.600 | 131.0 | 121.0 | 150.0 | 143.0 | 125.0 | 151.0 | 0.352 | | 0.606 | |  |
| LDL-c, *mg/dL* | 79.0 | 59.5 | 107.5 | 87.0 | 63.0 | 110.0 | 0.861 | 72.0 | 50.0 | 80.0 | 69.0 | 58.0 | 96.0 | 0.735 | | 0.937 | |  |
| HDL-c, *mg/dL* | 42.0 | 39.0 | 48.0 | 42.0 | 38.5 | 49.0 | 0.937 | 32.0 | 31.0 | 40.0 | 32.0 | 28.0 | 38.0 | 0.141 | | 0.211 | |  |
| Triglycerides, *mg/dL* | 152.0 | 93.5 | 213.0 | 131.0 | 99.0 | 241.0 | 0.278 | 138.0 | 97.0 | 192.0 | 183.0 | 101.0 | 205.0 | 0.091 | | 0.552 | |  |
| Glucose, *mg/dL* | 116.0 | 107.0 | 142.0 | 108.0 | 85.5 | 124.0 | 0.530 | 107.0 | 90.0 | 147.0 | 118.0 | 86.0 | 150.0 | 0.735 | | 0.606 | |  |
| ***Metabolism*** |  |  |  |  |  |  |  |  |  |  |  |  |  |  | |  | |  |
| Sirtuin 1, *ng/mL* | 0.0013 | 0.0012 | 0.0129 | 0.0012 | 0.0012 | 0.0012 | 0.208 | 0.0012 | 0.0012 | 0.0039 | 0.0012 | 0.0012 | 0.0040 | 0.180 | | 0.075 | |  |
| Sirtuin 3, *ng/mL* | 1.1 | 0.9 | 4.0 | 1.1 | 0.8 | 4.4 | 0.152 | 0.9 | 0.8 | 1.1 | 1.0 | 0.7 | 1.4 | 0.735 | | 0.219 | |  |
| sRAGE, *pg/mL* | 839.7 | 577.3 | 1146.2 | 924.7 | 767.6 | 1215.0 | 0.064 | 841.4 | 462.6 | 970.9 | 706.6 | 569.0 | 1280.0 | 0.735 | | 0.219 | |  |
| Leptin, *ng/mL* | 46.6 | 3.3 | 63.7 | 40.6 | 4.0 | 48.9 | 0.972 | 3.5 | 1.4 | 11.4 | 2.2 | 1.5 | 2.5 | 0.063 | | 0.143 | |  |
| Adiponectin, *μg/mL* | 7.2 | 6.1 | 11.9 | 7.4 | 6.3 | 11.8 | 0.600 | 9.9 | 8.6 | 13.5 | 9.7 | 7.6 | 11.9 | 0.091 | | 0.104 | |  |
| ***Endothelial function and inflammation*** |  |  |  |  |  |  |  |  |  |  |  |  |  |  | |  | |  |
| CD34^+^/KDR^+^, *%* | 0.19 | 0.18 | 0.33 | 0.24 | 0.16 | 0.30 | 0.254 | 0.27 | 0.20 | 0.34 | 0.16 | 0.08 | 0.27 | 0.144 | | 0.177 | |  |
| CD34^+^/KDR^+^, *cells/μL* | 16.29 | 11.69 | 26.74 | 14.21 | 11.88 | 22.32 | 0.203 | 16.57 | 12.71 | 28.91 | 8.88 | 5.25 | 17.30 | 0.080 | | 0.624 | |  |
| GM-CSF, *pg/mL* | 0.76 | 0.60 | 2.79 | 0.92 | 0.63 | 2.53 | 0.600 | 0.75 | 0.71 | 1.08 | 0.62 | 0.49 | 1.01 | 0.176 | | 0.452 | |  |
| MMP-9, *ng/mL* | 16.14 | 9.04 | 30.28 | 18.21 | 9.43 | 24.46 | 0.701 | 10.16 | 5.45 | 41.30 | 13.63 | 9.02 | 39.58 | 0.499 | | 0.322 | |  |
| VEGF, *pg/mL* | 0.85 | 0.85 | 1.45 | 0.85 | 0.85 | 0.85 | 0.273 | 0.85 | 0.85 | 4.88 | 0.85 | 0.85 | 106.40 | 0.109 | | **0.029** | |  |
| VCAM-1, *ng/mL* | 80.28 | 71.87 | 102.62 | 80.28 | 72.64 | 102.62 | 0.972 | 102.62 | 71.57 | 102.62 | 78.71 | 72.25 | 102.62 | 0.753 | | 0.751 | |  |
| Norepinephrine, *pg/mL* | 1256.60 | 1079.05 | 1526.80 | 1351.00 | 1148.40 | 1560.10 | 0.422 | 1315.40 | 1189.60 | 1627.80 | 1283.40 | 1199.80 | 1689.40 | 0.398 | | 0.405 | |  |
| hsCRP, *mg/L* | 1.3 | 0.8 | 2.1 | 1.2 | 0.8 | 4.4 | 0.350 | 1.7 | 0.7 | 2.9 | 1.6 | 0.9 | 1.8 | 0.735 | | 0.405 | |  |
| IL-1β, *pg/mL* | 10.1 | 3.3 | 12.8 | 14.3 | 8.7 | 45.3 | 0.249 | 12.7 | 1.4 | 19.9 | 10.3 | 8.4 | 24.5 | 0.735 | | 0.663 | |  |
| IL-6, *pg/mL* | 0.4 | 0.2 | 5.5 | 1.6 | 0.2 | 4.4 | 0.721 | 7.1 | 0.2 | 13.2 | 10.1 | 0.2 | 25.8 | 0.138 | | 0.188 | |  |
| IL-10, *pg/mL* | 6.1 | 3.1 | 8.3 | 1.2 | 0.0 | 6.5 | 0.101 | 7.6 | 2.6 | 9.1 | 3.0 | 0.9 | 7.0 | 0.866 | | 0.721 | |  |
| TNF-α, *pg/mL* | 19.1 | 13.2 | 27.2 | 22.4 | 15.6 | 32.2 | 0.422 | 26.6 | 14.2 | 37.7 | 39.0 | 20.9 | 49.4 | 0.128 | | 0.362 | |  |
| MCP-1, *pg/mL* | 93.77 | 82.87 | 122.25 | 100.20 | 89.44 | 119.90 | 0.972 | 76.45 | 69.91 | 105.10 | 102.60 | 76.94 | 118.40 | 0.499 | | 0.452 | |  |
| ***Apoptosis*** |  |  |  |  |  |  |  |  |  |  |  |  |  |  | |  | |  |
| Caspase 3, *ng/mL* | 2.27 | 1.05 | 2.96 | 2.44 | 1.26 | 4.71 | 0.463 | 2.13 | 1.31 | 3.51 | 1.55 | 1.41 | 2.00 | 0.499 | | 0.285 | |  |
| Caspase 9, *ng/mL* | 0.97 | 0.72 | 2.50 | 1.18 | 0.34 | 2.55 | 0.916 | 2.64 | 1.80 | 3.81 | 1.79 | 0.09 | 5.60 | 1.000 | | 0.905 | |  |
| Survivin, *pg/mL* | 50.00 | 36.00 | 116.50 | 31.00 | 22.00 | 48.50 | 0.075 | 45.00 | 11.00 | 72.00 | 32.00 | 22.00 | 79.00 | 0.866 | | 0.143 | |  |
| cIAP2, *ng/mL* | 1.49 | 0.77 | 1.99 | 1.18 | 0.91 | 2.01 | 0.382 | 1.44 | 1.00 | 1.73 | 1.54 | 1.02 | 2.29 | **0.018** | | 0.552 | |  |
| XIAP, *ng/mL* | 0.23 | 0.04 | 5.56 | 0.62 | 0.04 | 10.05 | 0.214 | 0.57 | 0.04 | 4.82 | 0.04 | 0.04 | 0.05 | 0.068 | | **0.047** | |  |
| Bcl-2, *ng/mL* | 0.85 | 0.23 | 2.63 | 0.85 | 0.16 | 1.76 | 0.859 | 0.64 | 0.33 | 1.35 | 1.95 | 0.52 | 3.06 | **0.043** | | 0.062 | |  |
| Cytochrome c, *pg/mL* | 3.94 | 2.00 | 64.59 | 2.00 | 2.00 | 47.84 | 0.859 | 2.00 | 2.00 | 46.25 | 2.00 | 2.00 | 32.63 | 1.000 | | 0.713 | |  |
| sTNFR2, *ng/mL* | 6.56 | 5.53 | 7.92 | 6.55 | 5.23 | 8.00 | 1.000 | 6.10 | 4.50 | 7.86 | 6.24 | 4.17 | 8.00 | 0.116 | | 0.321 | |  |
| S100A12, *pg/mL* | 1380.80 | 1018.40 | 2733.50 | 856.00 | 769.44 | 1565.20 | 0.087 | 870.40 | 751.12 | 1112.80 | 900.80 | 739.28 | 1384.80 | 0.612 | | 0.191 | |  |
| ***Endothelial progenitor cells mobilization*** |  |  |  |  |  |  |  |  |  |  |  |  |  |  | |  | |  |
| Angiopoietin 2, *pg/mL* | 228.10 | 202.50 | 318.60 | 277.00 | 207.10 | 395.15 | 0.249 | 301.70 | 272.90 | 394.90 | 332.70 | 254.30 | 391.10 | 0.866 | | 0.322 | |  |
| SCF, *pg/mL* | 165.4 | 135.6 | 194.1 | 148.9 | 116.9 | 213.9 | 0.861 | 140.8 | 116.9 | 195.0 | 163.1 | 120.2 | 213.2 | 0.310 | | 0.251 | |  |
