# Supplementary material for: The effects of resveratrol on endothelial progenitor cells and apoptosis biomarkers in postmenopausal women with chronic coronary heart disease: a randomized controlled trial
Source: Front Nutr. 2026 May 14;13:1814668. doi: 10.3389/fnut.2026.1814668 (PMC13215931; doi:10.3389/fnut.2026.1814668)
Supplement: Supplementary file 1 [file Data_Sheet_1.zip › Supplementary Table 1 to 3.docx]

**Supplementary Table 1.** Nutritional characteristics of the participants at the baseline and post-treatment.

| **Variables** | **Placebo (n = 13)** | | | | | |  | **Resveratrol (n = 7)** | | | | | |  |  | |
| --- | --- | --- | --- | --- | --- | --- | --- | --- | --- | --- | --- | --- | --- | --- | --- | --- |
|  | **Baseline** | | | **Post-intervention** | | |  | **Baseline** | | | **Post-intervention** | | |  | **Δ** | |
|  | **Median** | **IQR** | | **Median** | **IQR** | | ***p*** | **Median** | **IQR** | | **Median** | **IQR** | | ***p*** | ***p*** |  |
| *Dietary intake* |  |  |  |  |  |  |  |  |  |  |  |  |  |  |  |  |
| Energy, *kcal/d* | 1446.0 | 1124.0 | 1636.5 | 1149.0 | 837.0 | 1704.0 | 0.241 | 1625.0 | 1455.0 | 2161.0 | 1625.0 | 1257.0 | 1648.0 | 0.465 | 0.688 |  |
| Proteins, *%kcal* | 15.2 | 12.6 | 18.9 | 16.7 | 9.8 | 27.6 | 0.333 | 16.2 | 10.0 | 27.2 | 18.0 | 9.2 | 24.4 | 1.000 | 0.630 |  |
| Carbohydrates, *%kcal* | 58.1 | 49.0 | 64.8 | 51.7 | 46.5 | 66.9 | 0.878 | 47.6 | 40.2 | 67.2 | 58.6 | 51.9 | 70.6 | 0.144 | 0.335 |  |
| Fibers, *g* | 10.4 | 7.7 | 12.7 | 10.4 | 8.3 | 19.8 | 0.646 | 16.0 | 9.7 | 24.6 | 15.3 | 12.3 | 18.8 | 1.000 | 0.840 |  |
| Lipids, *%kcal* | 28.4 | 22.7 | 36.6 | 25.5 | 20.9 | 29.5 | 0.203 | 31.0 | 23.9 | 42.5 | 28.0 | 23.0 | 28.5 | 0.273 | 0.810 |  |
| Saturated fatty acids, *%kcal* | 9.3 | 7.9 | 11.4 | 7.5 | 6.1 | 9.4 | 0.333 | 10.7 | 8.8 | 14.1 | 9.5 | 8.5 | 12.2 | 0.273 | 0.748 |  |
| Monounsaturated fatty acids, *%kcal* | 8.8 | 5.4 | 11.0 | 6.5 | 3.2 | 8.7 | **0.037** | 10.0 | 5.9 | 16.9 | 7.6 | 5.9 | 10.0 | 0.144 | 0.748 |  |
| Polyunsaturated fatty acids, *%kcal* | 5.1 | 2.9 | 7.8 | 3.6 | 1.9 | 6.2 | 0.074 | 5.8 | 3.6 | 6.2 | 3.7 | 2.2 | 5.6 | 0.273 | 1.000 |  |
| *Trans* fatty acids, *%kcal* | 0.1 | 0.0 | 0.4 | 0.0 | 0.0 | 0.3 | 0.398 | 0.1 | 0.0 | 0.7 | 0.1 | 0.0 | 0.5 | 0.715 | 0.648 |  |
| *Anthropometry* |  |  |  |  |  |  |  |  |  |  |  |  |  |  |  |  |
| Body mass index, *kg/m^2^* | 30.4 | 26.1 | 32.2 | 30.0 | 26.7 | 33.3 | 0.239 | 26.0 | 20.1 | 27.7 | 25.5 | 20.2 | 27.9 | 0.396 | 0.219 |  |
| Skinfold sum, *mm* | 91.0 | 66.5 | 105.5 | 91.0 | 81.5 | 113.0 | 0.421 | 73.0 | 41.0 | 87.0 | 70.0 | 48.0 | 77.0 | 0.499 | 0.284 |  |
| Arm circumference, *cm* | 30.0 | 28.0 | 32.5 | 29.0 | 28.3 | 33.2 | 0.937 | 28.0 | 25.0 | 32.3 | 26.0 | 24.8 | 29.0 | 0.061 | 0.054 |  |
| Waist circumference, *cm* | 94.0 | 84.5 | 105.5 | 99.0 | 91.3 | 111.0 | 0.117 | 82.0 | 74.0 | 96.0 | 82.0 | 77.5 | 99.0 | 0.343 | 0.578 |  |
| Arm muscle circumference, *cm* | 22.4 | 21.6 | 24.7 | 21.8 | 20.5 | 24.6 | 0.382 | 21.3 | 20.1 | 24.4 | 20.7 | 19.7 | 23.3 | 0.236 | 0.578 |  |
| Fat-free mass, *kg* | 43.7 | 40.3 | 48.7 | 42.4 | 40.3 | 47.2 | 0.859 | 40.0 | 36.7 | 40.5 | 40.2 | 36.3 | 41.1 | 0.866 | 0.781 |  |
| Fat mass, *kg* | 22.9 | 19.5 | 29.5 | 22.7 | 21.1 | 29.5 | 0.255 | 20.3 | 12.9 | 21.4 | 18.5 | 13.5 | 20.6 | 0.612 | 0.250 |  |
| Body fat, *%* | 35.5 | 31.2 | 37.5 | 35.5 | 34.0 | 38.5 | 0.421 | 32.5 | 24.8 | 34.9 | 31.9 | 26.9 | 33.2 | 1.000 | 0.250 |  |
| *Physical activity** |  |  |  |  |  |  |  |  |  |  |  |  |  |  |  |  |
| Sedentary, *frequency and %* | 7.0 | 53.8 | | 7.0 | 53.8 | | N/A | 5.0 | 71.4 | | 5.0 | 71.4 | | N/A | N/A |  |
| Irregularly active, *frequency and %* | 2.0 | 15.4 | | 2.0 | 15.4 | | N/A | 1.0 | 14.3 | | 1.0 | 14.3 | | N/A | N/A |  |
| Active, *frequency and %* | 4.0 | 30.8 | | 4.0 | 30.8 | | N/A | 1.0 | 14.3 | | 1.0 | 14.3 | | N/A | N/A |  |
| Very active, *frequency and %* | 0.0 | 0.0 | | 0.0 | 0.0 | | N/A | 0.0 | 0.0 | | 0.0 | 0.0 | | N/A | N/A |  |

**Supplementary Table 2.** Mediation analysis.

| **Models (variable's Δ)** | **Direct effect (*c'*)** | ***a*** | ***SE_a_*** | ***b*** | ***SE_b_*** | **Indirect effect (*a*b*)** | **Sobel's *Z*** | ***p*** |  |
| --- | --- | --- | --- | --- | --- | --- | --- | --- | --- |
|  |  |  |  |  |  |  |  |  |  |
| **Sirtuin 1, *ng/mL*** |  |  |  |  |  |  |  |  |  |
| Resveratrol | 0.002 | N/A | N/A | N/A | N/A | N/A | N/A | N/A |  |
| Norepinephrine, *pg/mL* |  | 117.941 | 207.562 | **0.000016** | **0.000** | 0.001848 | 0.547 | 0.585 |  |
| Survivin, *pg/mL* |  | 108.630 | 126.715 | **0.000019** | **0.000** | 0.002013 | 0.789 | 0.430 |  |
| **Sirtuin 3, *ng/mL*** |  |  |  |  |  |  |  |  |  |
| Resveratrol | -0.557 | N/A | N/A | N/A | N/A | N/A | N/A | N/A |  |
| XIAP, *ng/mL* |  | -5.893 | 3.992 | **-0.143** | **0.019** | 0.843 | 1.449 | 0.147 |  |
| Adiponectin, *μg/mL* |  | -1.172 | 0.764 | **-0.388** | **0.100** | 0.455 | 1.423 | 0.154 |  |
| **CD34^+^/KDR^+^, *%*** |  |  |  |  |  |  |  |  |  |
| Resveratrol | **-0.249** | N/A | N/A | N/A | N/A | N/A | N/A | N/A |  |
| sTNFR2, *ng/mL* |  | -2.732 | 4.301 | **0.143** | **0.004** | -0.391 | -0.635 | 0.525 |  |
| HDL-c, *mg/dL* |  | -2.516 | 2.025 | **-0.030** | **0.007** | 0.075 | 1.242 | 0.214 |  |
| XIAP, *ng/mL* |  | -5.893 | 3.992 | **-0.011** | **0.003** | 0.065 | 1.369 | 0.171 |  |
| **cIAP2, *ng/mL*** |  |  |  |  |  |  |  |  |  |
| Resveratrol | -0.042 | N/A | N/A | N/A | N/A | N/A | N/A | N/A |  |
| Bcl-2, *ng/mL* |  | 1.090 | 0.610 | **0.216** | **0.063** | 0.235 | 1.585 | 0.113 |  |
| **Bcl-2, *ng/mL*** |  |  |  |  |  |  |  |  |  |
| Resveratrol | -0.004 | N/A | N/A | N/A | N/A | N/A | N/A | N/A |  |
| S100A12, *pg/mL* |  | 1023.896 | 604.395 | **0.001** | **0.000** | 1.024 | 1.670 | 0.095 |  |
| cIAP2, *ng/mL* |  | 0.193 | 0.206 | **1.549** | **0.403** | 0.299 | 0.910 | 0.363 |  |
| MCP-1, *pg/mL* |  | 17.264 | 16.485 | **0.014** | **0.005** | 0.242 | 0.981 | 0.327 |  |
| **Survivin, *pg/mL*** |  |  |  |  |  |  |  |  |  |
| Resveratrol | -34.575 | N/A | N/A | N/A | N/A | N/A | N/A | N/A |  |
| IL-1β, *pg/mL* |  | -22.802 | 24.843 | **-4.815** | **0.528** | 109.792 | 0.913 | 0.361 |  |
| Leptin, *ng/mL* |  | -6.995 | 12.419 | **-3.270** | **0.986** | 22.874 | 0.555 | 0.579 |  |
| Angiopoietin 2, *pg/mL* |  | -16.007 | 59.426 | **-0.659** | **0.223** | 10.549 | 0.268 | 0.789 |  |
| **XIAP, *ng/mL*** |  |  |  |  |  |  |  |  |  |
| Resveratrol | -3.968 | N/A | N/A | N/A | N/A | N/A | N/A | N/A |  |
| Sirtuin 3, *ng/mL* |  | 0.740 | 0.698 | **-5.476** | **0.641** | -4.052 | -1.052 | 0.298 |  |
| Adiponectin, *μg/mL* |  | -1.172 | 0.764 | **-1.880** | **0.599** | 2.203 | 1.378 | 0.168 |  |
| GM-CSF, *pg/mL* |  | -0.037 | 0.484 | **2.081** | **0.879** | -0.077 | 0.076 | 0.939 |  |
| **Caspase 9, *ng/mL*** |  |  |  |  |  |  |  |  |  |
| Resveratrol | **-7.152** | N/A | N/A | N/A | N/A | N/A | N/A | N/A |  |
| hsCRP, *mg/L* |  | -0.885 | 0.852 | **8.320** | **1.007** | -7.363 | -1.031 | 0.303 |  |
| TNF-α, *pg/mL* |  | 28.819 | 16.510 | **0.321** | **0.052** | 9.251 | 1.779 | 0.075 |  |
| age, *years* |  | N/A | N/A | **1.033** | **0.255** | N/A | N/A | N/A |  |
| **Caspase 3, *ng/mL*** |  |  |  |  |  |  |  |  |  |
| Resveratrol | -1.225 | N/A | N/A | N/A | N/A | N/A | N/A | N/A |  |
| **Cytochrome c, *pg/mL*** |  |  |  |  |  |  |  |  |  |
| Resveratrol | 18.913 | N/A | N/A | N/A | N/A | N/A | N/A | N/A |  |
| Body fat, *%* |  | -0.854 | 0.919 | **14.789** | **4.497** | -12.630 | -0.894 | 0.371 |  |
| Sirtuin 1, *ng/mL* |  | 0.006 | 0.005 | **-2041.741** | **902.627** | -12.250 | -1.060 | 0.289 |  |

**Supplementary Table 3.** Effects of resveratrol on circulating sirtuins, EPCs, and apoptosis biomarkers adjusted by baseline HDL-c and S100A12.

| **Models (variable's Δ)** | **Adjusted R^2^** | ***B*** | ***SE*** | ***p*** | **95% CI** | |
| --- | --- | --- | --- | --- | --- | --- |
|  |  |  |  |  | **Lower** | **Higher** |
| **Sirtuin 1, *ng/mL*** | 0.013 |  |  |  |  |  |
| Resveratrol |  | 0.01200 | 0.00700 | 0.096 | -0.002 | 0.026 |
| Baseline HDL-c, *mg/dL* |  | 0.00000 | 0.00000 | 0.381 | -0.001 | 0.001 |
| Baseline S100A12, *pg/mL* |  | 0.00000 | 0.00000 | 0.333 | 0.000 | 0.000 |
| **Sirtuin 3, *ng/mL*** | 0.179 |  |  |  |  |  |
| Resveratrol |  | -0.515 | 0.909 | 0.579 | -2.441 | 1.411 |
| Baseline HDL-c, *mg/dL* |  | -0.067 | 0.066 | 0.324 | -0.207 | 0.073 |
| Baseline S100A12, *pg/mL* |  | -0.001 | 0.000 | **0.033** | **-0.002** | **0.000** |
| **CD34^+^/KDR^+^, *%*** | 0.006 |  |  |  |  |  |
| Resveratrol |  | -1.406 | 0.837 | 0.113 | -3.182 | 0.369 |
| Baseline HDL-c, *mg/dL* |  | -0.072 | 0.061 | 0.256 | -0.201 | 0.057 |
| Baseline S100A12, *pg/mL* |  | 0.000 | 0.000 | 0.248 | -0.001 | 0.000 |
| **cIAP2, *ng/mL*** | 0.117 |  |  |  |  |  |
| Resveratrol |  | 0.436 | 0.276 | 0.133 | -0.149 | 1.021 |
| Baseline HDL-c, *mg/dL* |  | 0.038 | 0.020 | 0.075 | -0.004 | 0.081 |
| Baseline S100A12, *pg/mL* |  | 0.000 | 0.000 | 0.573 | 0.000 | 0.000 |
| **Bcl-2, *ng/mL*** | 0.121 |  |  |  |  |  |
| Resveratrol |  | 1.503 | 0.865 | 0.102 | -0.331 | 3.337 |
| Baseline HDL-c, *mg/dL* |  | 0.078 | 0.063 | 0.232 | -0.055 | 0.212 |
| Baseline S100A12, *pg/mL* |  | 0.000 | 0.000 | 0.520 | -0.001 | 0.001 |
| **Survivin, *pg/mL*** | 0.076 |  |  |  |  |  |
| Resveratrol |  | 310.317 | 173.195 | 0.092 | -56.840 | 677.474 |
| Baseline HDL-c, *mg/dL* |  | 24.316 | 12.595 | 0.071 | -2.385 | 51.017 |
| Baseline S100A12, *pg/mL* |  | 0.017 | 0.082 | 0.840 | -0.157 | 0.190 |
| **XIAP, *ng/mL*** | 0.299 |  |  |  |  |  |
| Resveratrol |  | 3.679 | 4.933 | 0.467 | -6.778 | 14.137 |
| Baseline HDL-c, *mg/dL* |  | 0.703 | 0.359 | 0.068 | -0.057 | 1.464 |
| Baseline S100A12, *pg/mL* |  | 0.006 | 0.002 | **0.029** | **0.001** | **0.011** |
| **Caspase 9, *ng/mL*** | -0.001 |  |  |  |  |  |
| Resveratrol |  | -5.571 | 8.146 | 0.504 | -22.840 | 11.698 |
| Baseline HDL-c, *mg/dL* |  | -0.416 | 0.592 | 0.492 | -1.672 | 0.840 |
| Baseline S100A12, *pg/mL* |  | 0.004 | 0.004 | 0.285 | -0.004 | 0.012 |
| **Caspase 3, *ng/mL*** | 0.029 |  |  |  |  |  |
| Resveratrol |  | -2.067 | 1.470 | 0.179 | -5.184 | 1.050 |
| Baseline HDL-c, *mg/dL* |  | -0.013 | 0.107 | 0.903 | -0.240 | 0.213 |
| Baseline S100A12, *pg/mL* |  | -0.001 | 0.001 | 0.165 | -0.002 | 0.000 |
| **Cytochrome c, *pg/mL*** | 0.155 |  |  |  |  |  |
| Resveratrol |  | -48.436 | 30.334 | 0.130 | -112.740 | 15.868 |
| Baseline HDL-c, *mg/dL* |  | -2.097 | 2.206 | 0.356 | -6.773 | 2.579 |
| Baseline S100A12, *pg/mL* |  | -0.036 | 0.014 | **0.025** | **-0.066** | **-0.005** |
